# Symptoms predicting remission after divalproex augmentation with olanzapine in partially nonresponsive patients experiencing mixed bipolar I episode: a post-hoc analysis of a randomized controlled study

**DOI:** 10.1186/1756-0500-3-276

**Published:** 2010-11-02

**Authors:** John P Houston, Jennifer L Gatz, Elisabeth K Degenhardt, Hassan H Jamal

**Affiliations:** 1 US Medical Neuroscience, Lilly USA, LLC; Drop Code 4133, Indianapolis, IN 46285 USA; 2 Department of Psychiatry, Indiana University School of Medicine, Indianapolis, IN 46202 USA

## Abstract

**Background:**

Rating scale items in a 6-week clinical trial of olanzapine versus placebo augmentation in patients with mixed bipolar disorder partially nonresponsive to ≥14 days of divalproex monotherapy were analyzed to characterize symptom patterns that could predict remission. At baseline, the two treatment groups were similar.

**Findings:**

Factor analysis with Varimax rotation was performed *post hoc *on baseline items of the 21-Item Hamilton Depression Rating Scale (HDRS-21) and Young Mania Rating Scale (YMRS). Backwards-elimination logistic regression ascertained factors predictive of protocol-defined endpoint remission (HDRS-21 score ≤ 8 and YMRS score ≤ 12) with subsequent determination of optimally predictive factor score cutoffs.

Factors for Psychomotor activity (YMRS items for elevated mood, increased motor activity, and increased speech and HDRS-21 agitation item) and Guilt/Suicidality (HDRS-21 items for guilt and suicidality) significantly predicted endpoint remission in the divalproex+olanzapine group. No factor predicted remission in the divalproex+placebo group. Patients in the divalproex+olanzapine group with high pre-augmentation psychomotor activity (scores ≥10) were more likely to remit compared to those with lower psychomotor activity (odds ratio [OR] = 3.09, 95% confidence interval [CI] = 1.22-7.79), and patients with marginally high Guilt/Suicidality (scores ≥2) were less likely to remit than those with lower scores (OR = 0.37, 95% CI = 0.13-1.03). Remission rates for divalproex+placebo vs. divalproex+olanzapine patients with high psychomotor activity scores were 22% vs. 45% (p = 0.08) and 33% vs. 48% (p = 0.29) for patients with low Guilt/Suicidality scores.

**Conclusions:**

Patients who were partially nonresponsive to divalproex treatment with remaining high vs. low psychomotor activity levels or minimal vs. greater guilt/suicidality symptoms were more likely to remit with olanzapine augmentation.

**Trial Registration:**

ClinicalTrials.gov; http://clinicaltrials.gov/ct2/show/NCT00402324?term=NCT00402324&rank=1, Identifier: NCT00402324

## Background

Variable pharmacologic treatment response has led to analyses of associations with previous clinical course [1-4] and baseline clinical characteristics [1,3,5,6] to develop tailored therapies. A *post hoc *factor analysis of baseline components from the 21-item Hamilton Depression Rating Scale (HDRS-21 [7]) and the Young Mania Rating Scale (YMRS) [8] in bipolar I disorder patients with a current mixed episode characterized specific baseline mood symptoms which potentially predicted remission with treatment augmentation. Data were from a 6-week, double-blind, placebo-controlled trial, of olanzapine (OLZ) augmentation in patients with inadequate response to divalproex (DPX). The primary efficacy endpoint for the original clinical trial was a change in YMRS and HDRS-21 from baseline across 6 weeks by therapy. The primary study was reviewed and approved by the institutional review board at each site, and was conducted in accordance with the International Conference on Harmonisation Good Clinical Practice guidelines. Verbal and written informed consent was obtained from all subjects prior to participation. The study, which demonstrated a statistically significant difference between overall treatment groups in improvement over 6 weeks (in both YMRS and HDRS-21 scores), primary endpoints for the original clinical trial, was not adequately powered to show a difference in treatment arms for dual remission rate (31% vs. 26%, p = .437 for OLZ augmentation vs. continued DPX monotherapy) [9]. Part of the rationale for performing the present analysis was to determine whether baseline symptoms effectively determined sub-groups of patients more likely to remit with olanzapine augmentation vs. continued DPX monotherapy, given the low overall remission rate.

## Methods

Outpatients aged 18 to 60 years with bipolar I disorder, current mixed episode (with or without psychotic features), as defined by the *Diagnostic and Statistical Manual of Mental Disorders*, 4th edition, (DSM-IV-TR; diagnostic criteria 296.6x) were included. Those (n = 202) with inadequate response to ≥14 days of oral DPX monotherapy (blood levels 75 to 125 μg/mL) were randomly assigned to 6 weeks of placebo (PLA; [*n *= 101]) or OLZ (mean modal daily dose 14.6 mg; *n *= 101]) augmentation of DPX treatment. Inadequate response was defined as a HDRS-21 score ≥16 and a YMRS score ≥16. Clinical Global Impressions of Severity (CGI-S) for Bipolar Illness scale data were also collected at baseline. Study details are available elsewhere [9]. Briefly, baseline characteristics of the two treatment groups at randomization were similar in the DPX+PLA vs. DPX+OLZ study arms: 55% vs. 46% white, 29% vs. 38% African American, and 43% vs. 40% male with mean HDRS-21 scores of 21.9 vs. 22.5 and YMRS scores of 20.4 vs. 21.4 [9].

Factor analysis is a statistical technique that combines individual variables varying in the same manner or direction into representative summary factors. Factor analysis permits a reduction in the dimensionality of the original data and the identification of clinically-defined patient subgroups. Factor analysis with Varimax rotation was performed on HDRS-21 and YMRS items, obtained prior to augmentation therapy [10]. Items with factor-loading scores ≥0.4 were included in the one factor for which they had the highest factor-loading score. Items with a negative loading factor were reverse-scored. Only factors with an eigenvalue > 1 were included in the analyses. Pearson correlation coefficients between these factors and CGI-S scores were determined at baseline.

Backwards-elimination logistic regression models were run for DPX+OLZ and DPX+PLA separately for remission (HDRS-21 score ≤ 8 and YMRS score ≤ 12) at 6 weeks. Investigative site location (US vs. Puerto Rico) and all factors derived from the factor analysis were included in the initial model as potential predictors. Variables were eliminated from the model at the 0.05 level of statistical significance.

Several potential cut-off scores (median, median ± 1, 2) for each factor that were significant in the logistic regression models were investigated as predictors of remission. T-tests of each of these potential cut-off scores against remission were performed to find the factor cut-off score that best divided the remitters from the non-remitters within that treatment group based on statistical significance. Sensitivity, specificity, positive predictive value (PPV), and negative predictive value (NPV) were also determined. Logistic regression models were performed using the categorical factor cut-off scores to predict ultimate remission.

Analyses of the YMRS and HRDS-21 total scores over time in sub-groups separated by initial factor cut-off scores utilized the mixed model repeated measures (MMRM) approach over 6 weeks. The models included the fixed, categorical effects of factor cut-off score, visit, baseline total score, investigative site, and visit-by-factor cut-off interaction. The optimal within-subject covariance matrix in each MMRM model was determined by Bayesian Information Criterion after testing the following options: unstructured, toeplitz, auto-regressive, and compound-symmetric. SAS version 9.1.3 was used for all analyses.

Remission rates for statistically significant factors in patients sub-grouped by optimal cut-off factor scores were compared between DPX+PLA and DPX+OLZ groups by Fisher's exact test.

## Results

Using principal factor analysis with Varimax rotation, the 32 individual HDRS-21 and YMRS items were reduced into 11 factors with eigenvalues > 1 as shown in Table 1. These 11 factors explained 62% of the total variance of the baseline HDRS-21 and YMRS item scores. Eigenvalues did not drop substantially in value across the sequence. Psychomotor activity (Factor 2), Work impairment/somatization (Factor 4), Irritability (Factor 5), Guilt/Suicidality (Factor 8), and Appearance/Gastrointestinal symptoms (Factor 9) correlated significantly with CGI-S (Table 1). Sleep disturbance (Factor 1) contributed significantly to the total scores from HDRS-21 and YMRS items, but did not correlate significantly with CGI-S. In a logistic regression model that included these 11 factors at baseline and investigative site as independent variables (and remission as the dependent variable), only Psychomotor activity and Guilt/Suicidality and investigative site were significant predictors of remission in DPX+OLZ-treated patients (Table 2). In the DPX-PLA treated patients, no factors remained in the backwards elimination model at a 0.05 significance level.

**Table 1:** Factor Analysis [12] of Mania and Depression Symptoms

Factor No. Name	Scale Items	Mean Contribution To HDRS-21 + YMRS Total Score	Eigenvalue	Pearson Correlation Coefficient with CGI (p value)
1. Sleep disturbance	YMRS 4 Sleep HDRS-21 4 Early Insomnia HDRS-21 5 Middle Insomnia HDRS-21 6 Late Insomnia	13.90%	2.82	0.073 (0.301)
2. Psychomotor activity	YMRS 1 Elevated Mood YMRS 2 Increased Psychomotor Activity YMRS 6 Rapid Speech HDRS-21 9 Agitation	19.70%	2.53	0.253 (< .001)*
3. Insight	YMRS 11 Lack of Insight HDRS 17 Lack of Insight	0.40%	2.26	-0.043 (0.541)
4. Work impairment/Somatization	HDRS-21 7 Work and Activities HDRS-21 11 Somatic Anxiety HDRS-21 13 General Somatic Symptoms	12.80%	2.10	0.293 (< .001)*
5. Irritability	YMRS 5 Irritability YMRS 9 Disruptive Aggressive Behavior HDRS-21 1 Depressed Mood	20.70%	1.83	0.181 (< .01)*
6. Variation/Derealization/Obsession	HDRS-21 18b Diurnal Variation HDRS-21 19 Depersonalization and Derealization HDRS-21 21 Obsessional and Compulsive Symptoms	2.90%	1.70	0.073 (0.303)
7. Sexuality	YMRS 3 Sexual Interest HDRS-21 14 Genital Symptoms (reverse)	3.80%	1.48	-0.120 (0.089)
8. Guilt/Suicidality	HDRS-21 2 Feelings of Guilt HDRS-21 3 Suicide	4.80%	1.38	0.256 (< .001)*
9. Appearance/GI	YMRS 10 Appearance HDRS-21 12 Somatic Symptoms (gastrointestinal) HDRS-21 16 Loss of Weight	3.10%	1.28	0.298 (< .001)*
10. Thought disorder	YMRS 8 Content HDRS-21 20 Paranoid Symptoms	6.40%	1.25	0.012 (0.871)
11. Retardation/Hypochondriasis	HDRS-21 8 Psychomotor Retardation (reverse) HDRS-21 15 Hypochondriasis	9.90%	1.08	0.118 (0.094)

* Statistically-significant (p < .05). GI: Gastrointestinal

**Table 2:** Details of Two Factors Predictive of Remission with Olanzapine Augmentation of Divalproex Partially Nonresponsive Patients.

Factor No:	CONTINUOUS VARIABLE ANALYSIS			CATEGORICAL VARIABLE ANALYSIS							
	
	Odds Ratio	95% CI	P value	Score Cutoff	Odds Ratio	95% CI	P value	Sensitivity %	Specificity%	PPV %	NPV %
2. Psychomotor activity	1.18	1.01 - 1.38	0.034	10	3.09	1.22 - 7.79	0.017	54.84	69.57	44.70	77.40
8. Guilt/Suicidality	0.61	0.39 - 0.94	0.025	2	0.37	0.13 - 1.03	0.056	35.48	82.61	47.80	74.01

The logistic regression models also included investigative site location (USA vs. Puerto Rico)

Further analysis of Psychomotor activity and Guilt/Suicidality factors revealed that baseline scores below 10 and 2, respectively, best separated patients who achieved remission from those who did not (non-remitters) with DPX+OLZ treatment. Logistic regression models using these categorical cut-offs showed that those with Psychomotor activity (Factor 2) scores ≥10 were more likely to remit at endpoint (p = 0.02). Those with Guilt/Suicidality (Factor 8) scores ≥2 were less likely to remit at endpoint, although this relationship did not quite reach significance (p = 0.06). The sensitivity, specificity, PPV, and NPV of these cut-off scores are shown in Table 2. As defined by these scores, only 22% of patients without higher levels of psychomotor activity and 26% of patients with even modest levels of suicidality or guilt feelings remaining after non-response to DPX treatment for at least 2 weeks remitted with OLZ augmentation.

**Figure 1:**
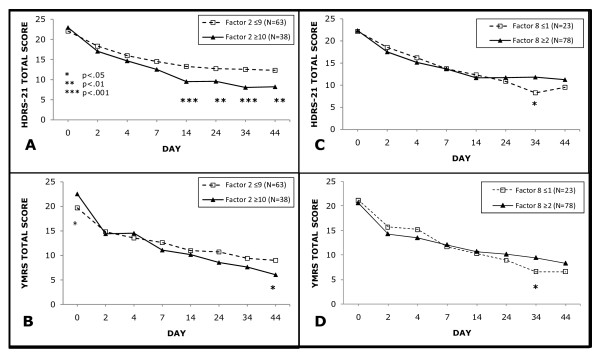
**Total HDRS-21 (A and C) and YMRS (B and D) scores in sub-categorized patients**. Patients were sub-categorized by cut-off scores for baseline Psychomotor activity (Factor 2) and Guilt/suicidality (Factor 8) during olanzapine augmentation of divalproex-partially nonresponsive patients. HDRS-21: 21-Item Hamilton Depression Rating Scale; YMRS: Young Mania Rating Scale.

In an MMRM model of changes in HDRS-21 and YMRS total scores over time, those with Psychomotor activity (Factor 2) scores ≥10 had significantly lower totals on the HDRS-21 score from 2 weeks post-randomization onwards (Figure 1A) and a significantly lower YMRS score at 6 weeks, despite a significantly higher YMRS score at baseline (Figure 1B). For Guilt/Suicidality (Factor 8), significant outcome differences were observed only at approximately 5 weeks post-randomization for HDRS-21 and YMRS scores (Figures 1C and 1D).

Remission rates in the DPX+PLA vs. DPX+OLZ groups were 21.9% vs. 44.7%, p = 0.08 for patients with high psychomotor activity (score ≥10) and 33.3% vs. 47.8% (p = 0.29) for patients with low Guilt/Suicidality (score < 2).

The categorical cut-off scores for Psychomotor activity (Factor 2) and Guilt/Suicidality (Factor 8) were also compared to the endpoint CGI-S score to evaluate their association with a measure of remission not based on YMRS or HDRS-21. Both of these categorical cut-offs in the OLZ treatment group were associated with a nearly half-point difference in the endpoint CGI-S scale though these relationships did not reach statistical significance (Psychomotor activity [Factor 2] < 10: mean CGI-S = 3.11 versus Psychomotor activity ≥10 mean CGI-S = 2.68, p = .08; Guilt/Suicidality (Factor 8) < 2, mean CGI-S = 2.57 versus Guilt/Suicidality ≥2, mean CGI-S = 3.06, p = .05).

## Discussion

We reduced 32 items of the HDRS-21 and YMRS at baseline into 11 factors, of which 2 factors predicted remission at 6 weeks following OLZ augmentation in patients previously partially nonresponsive to DPX monotherapy. Patients with greater baseline Psychomotor activity (Factor 2 ≥10) were more likely to remit with OLZ augmentation. These patients had lower final YMRS and HDRS-21 scores after 6 weeks of OLZ augmentation despite significantly higher YMRS and similar HDRS-21 scores at baseline. In contrast, patients with even marginally high Guilt/Suicidality (Factor 8≥2) were less likely to remit than those with lower levels of symptomatology. Guilt/Suicidality (Factor 8) correlated significantly with CGI-S scores despite a relatively low contribution to overall HDRS-21 and YMRS total scores (Table 1).

These findings along with relatively higher (although statistically nonsignificant) remission rates for high psychomotor activity and low Guilt/Suicidality groups with OLZ augmentation vs. continued DPX treatment alone suggest that these subgroups of patients may be better candidates for OLZ augmentation after inadequate response to DPX monotherapy. While Psychomotor activity and Guilt/suicidality had only moderate PPVs for remission (44.7% and 47.8%, respectively), in part related to the low overall rate of remission, they both had high NPVs (77.4% and 74.0%, respectively). Such predictors could increase the overall remission rate with OLZ augmentation through pre-treatment exclusion of patients likely to be non-remitters.

It is difficult to compare factors from our analysis with others in the literature because our population was restricted to bipolar disorder patients experiencing a mixed state. The factors we identified may be related to the presence of substantial depressive and manic symptoms. Also, a greater number of eigenvalues resulted from lack of at clear drop in eigenvalue magnitude between factors that would have allowed the elimination of factors associated with lower eigenvalues. Additionally, the methodology of other analyses differs from ours. One analysis developed a bivariate symptom rating scale based on 2 factors identified from a principal factor analysis in a retrospective study in which patients completed a symptom questionnaire based on their last manic episode [11]. Another analysis that used principal components factor analysis of HDRS-21 and YMRS scores in a group of patients with bipolar disorder who had experienced either mixed or manic states, yielded 5 factors corresponding to depression, dysphoria, hedonism, psychosis, and activation [10].

## Limitations

The likelihood of a type I error exists due to multiple testing (individual item analysis). Results cannot be generalized to other settings for characterization of baseline symptoms which predicted overall remission after OLZ augmentation. Although YMRS and HDRS-21 scales were used to define both predictors and outcomes, baseline factors composed of YMRS and HDRS-21 individual items defined predictors, and YMRS and HDRS-21 totals defined outcomes. YMRS and HDRS-21 measures used for predictors vs. outcomes were at different time points separated by up to six weeks. CGI-S scores also showed similar differences in endpoint changes from baseline when patients were sub-classified according to the categorical factor cut-off scores. The percent contribution of Factors 2 and 8 to total HDRS-21 and YMRS scores at baseline were 20% and 5%, respectively (Table 1). Since the study was not adequately powered to show a remission rate difference in treatment arms based upon the above cut-off scores, comparison of remission rates between treatments in high Psychomotor activity and low Suicidality/Guilt subgroups is not fully conclusive. While another limitation is that backwards elimination logistic regression is inferior to hypothesis-driven modeling, this exploratory *post-hoc *analysis sought the best predictive model using newly generated factors as predictors. Because no specific hypotheses regarding the factors were being tested, backwards elimination model building was an appropriate strategy.

## Conclusions

In this *post hoc *analysis of patients with bipolar disorder mixed episodes, factor analysis suggested two groups of HDRS-21 and YMRS items that predict remission in patients receiving OLZ augmentation following an inadequate response to DPX. Patients were more likely to remit with OLZ augmentation vs. continued DPX monotherapy if they scored lower on items related to guilt and suicidality and higher on items related to psychomotor activity.

## List of abbreviations

**CGI-S**: Clinical Global Impressions of Severity; **95% CI**: 95% confidence interval; **DPX**: divalproex; **HDRS-21**: 21-Item Hamilton Depression Rating Scale; **NPV**: negative predictive value; **OLZ**: olanzapine; **PLA**: placebo; **PPV**: positive predictive value; **YMRS**: Young Mania Rating Scale.

## Competing interests

Authors are employees of Lilly USA, LLC, and stockholders of Eli Lilly and Company.

## Authors' contributions

JPH, EKD and HHJ were involved in the original study protocol. JPH conceived the *post-hoc *study and participated in its analyses and interpretation. JLG performed the statistical analysis. HHJ drafted/updated the manuscript. All read, provided input for, and approved the final manuscript.
